# Variable Fall Climate Conditions on Carbon Assimilation and Spring Phenology of Young Peach Trees

**DOI:** 10.3390/plants9101353

**Published:** 2020-10-13

**Authors:** Brian T. Lawrence, Juan Carlos Melgar

**Affiliations:** 1 Department of Plant and Environmental Sciences, Clemson University, 105 Collings Street BRC 204, Clemson, SC 29634, USA; 2 Department of Plant and Environmental Sciences, Clemson University, 105 Collings Street BRC 218, Clemson, SC 29634, USA; jmelgar@clemson.edu

**Keywords:** deciduous fruit trees, climate change, photosynthesis, senescence, water deficit, sap flow, bloom timing

## Abstract

Variable fall temperature and moisture conditions may alter leaf senescence of deciduous fruit trees, influencing carbon assimilation before dormancy and phenology the following spring. This study explored gas exchange of young peach trees (*Prunus persica* (L.) Batsch) when senescence proceeded normally or was delayed during the fall under two soil moisture treatments: Well-irrigated trees or water deficit. Results showed leaf carbon assimilation was similar between the senescence treatments, but whole tree assimilation was estimated to be greater in delayed senescence trees compared to normal senescence trees based on timing of defoliation and total leaf area. The effect of soil moisture on carbon assimilation was not consistent between years. Delayed sap flow and bloom time resulted as a consequence of delayed senescence the previous fall, but soil moisture did not affect spring phenology.

## 1. Introduction

The impact of climate change on tree phenology has received increasing attention over the last 20 years [1,2,3,4]. Most studies which address variable climatic conditions and climate change on deciduous trees focus on how cold deprivation and the reduction of winter chill affect carbohydrate reserves during dormancy and spring carbon dynamics [5,6], and impact production [7,8]. However, annual variability of seasonal environmental conditions such as warmer-than-average temperatures [9,10,11] and moderate droughts [12,13] have been reported to advance or delay the onset of leaf senescence. Specifically, a delay of senescence during the fall may provide additional time for photosynthetically active leaves, thereby increasing carbon assimilation (*A*) and carbohydrate storage prior to leaf abscission [14], and increasing the annual productivity of future growing seasons [12,15]. However, how *A* is affected by variable climate conditions during fall and how senescence influences spring phenology are still poorly understood [12,16].

Fruit growers often apply water deficit during the postharvest period especially in regions where water is a limited resource. While some studies show the water-saving benefit of deficit irrigation strategies during the postharvest season for multiple fruit tree species including peach [17] and almond [18], other studies have identified the early postharvest stage as one of the most critical periods when water stress can affect yield [19,20]. Despite cultural practices that mitigate environmental stresses, many of the most productive fruit regions in the world are located in areas that are especially sensitive to climate patterns that bring abnormal temperature and precipitation such as El Niño/La Niña Southern Oscillation, the Pacific Decadal Oscillation, or the Indian Ocean Dipole [21,22,23,24]. Certain phases of these patterns result in warm and dry conditions that can last for an entire season, consecutive years, and even decades [21], altering the leaf phenology and length of growing seasons [12].

Variable environmental conditions during fall, at times exacerbated by cultural practices, are known to alter nutrient resorption and storage [25], and their influence on leaf *A* could also affect carbohydrate storage. Carbohydrate accumulation prior to dormancy is critical for deciduous trees, as they rely on carbohydrate reserves for budbreak and growth resumption in spring [16], and an increase of whole tree carbon assimilation (*A_tree_*) during fall as a consequence of delayed senescence (DS) could contribute to these reserves. However, DS results in the reduction of chill hour accumulation leading to erratic phenology patterns, such as bloom timing in spring [26,27]. We hypothesized that young peach trees that experienced DS and/or were well-watered (100% ET) during fall would have higher *A* than trees that experienced normal senescence (NS) and/or the trees that received 50% the well-watered amount (50% ET). We also hypothesized DS and lack of moisture during fall may delay the onset of sap flow movement and bloom timing at the beginning of spring. Therefore, the goal of this research was to understand *A* during fall of trees with different senescence timing and soil moisture conditions, and its effect in spring phenology. With this purpose, this study had the following objectives—(1) measure *A* of trees grown at temperature regimes inducing two senescence conditions and under two irrigation regimes throughout the fall season; and (2) monitor dormancy break by measuring flower development and initiation of sap flow in spring.

## 2. Results

### 2.1. Carbon Assimilation

**Figure 1:**
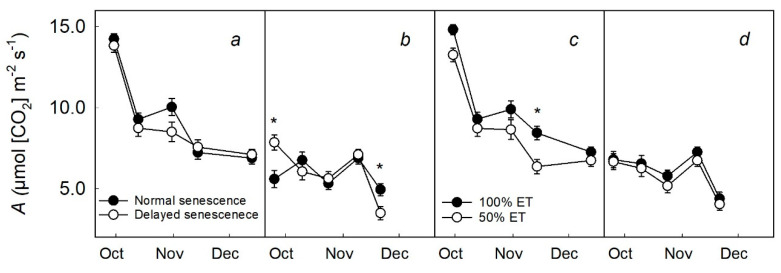
Average leaf CO_2_ assimilation (*A*) of young peach trees (*n* = 24) according to: (i) Senescence: trees in 2016 (**a**) and 2017 (**b**) grown under ambient fall temperatures (normal senescence, filled circles) or grown under the greenhouse environment, which resulted in a delay of senescence (delayed senescence, unfilled circles) and (ii) soil moisture: Trees in 2016 (**c**) and 2017 (**d**) that were well-watered (100% ET, filled circles) or water-deficient trees (50% ET, unfilled circles). Error bars represent ±standard error of the mean while asterisks show statistical differences (*p* ≤ 0.05) between the two treatments.

Leaf assimilation rates were similar for both DS and NS trees across dates during the fall of 2016 (Figure 1a) and 2017 (Figure 1b). Throughout the 2016 season, trees receiving 100% ET had higher *A* than in 50% ET trees, and the two treatments tended to separate based on standard error (Figure 1c), but rates were similar during 2017 measurements between dates (Figure 1d).

### 2.2. Leaf Count and A_tree_ during Senescence

**Figure 2:**
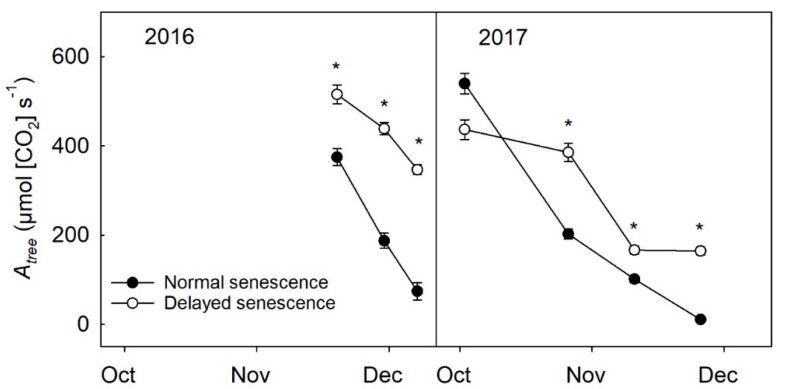
Estimation of whole tree assimilation (*A_tree_*) of normal senescence trees (filled circles) and delayed senescence trees (unfilled circles) during the fall of 2016 (*n* = 16) and 2017 (*n* = 12). The *A_tree_* estimate is calculated from an averaged assimilation value, leaf area, and leaf number per tree. Error bars represent ±standard error of the mean while asterisks show statistical differences (*p* ≤ 0.05) between the two treatments.

Compared to NS trees, DS trees kept in the warmer, greenhouse environment showed a delay of senescence; when leaf abscission began, the rate of abscission was significantly greater on NS trees than DS trees in 2016 (*p* ≤ 0.001) and 2017 (*p* ≤ 0.001), and approximately one-quarter of the leaves on DS trees never fully turned yellow or abscised before placing them outside during December both years. Soil moisture also resulted in significant differences in leaf abscission in 2016 (*p* ≤ 0.01) and 2017 (*p* ≤ 0.01), with 100% ET trees having a slower rate of defoliation than 50% ET trees in both years (data not shown), although these differences were minor compared to the differences observed between NS and DS trees. An estimation of *A_tree_* revealed DS trees had greater *A_tree_* than NS trees during the period of senescence and abscission in 2016 (*p* ≤ 0.001) and in 2017 (*p* ≤ 0.001) (Figure 2).

### 2.3. Biomass and Spring Phenology

Normal senescence trees had a higher root to shoot (R;S) ratio than DS trees in 2017. In 2018, differences were not significant, but the R:S ratio was numerically higher for NS trees. In 2018, the R:S ratios increased for all treatments compared to the 2017 values. There was no interaction between the senescence and soil moisture treatments either year (Table 1). 

**Figure 3:**
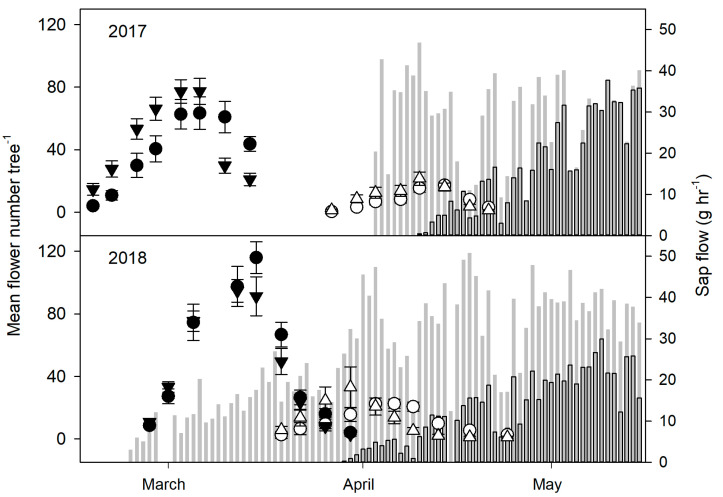
Mean number of spring flowers per tree at full bloom and sap flow in 2017 (*n* = 16) and 2018 (*n* = 8). The timing and amount of flowering of trees that had a normal senescence (filled symbols) was earlier and higher respectively compared to those that had a delayed senescence (open symbols), but similar between 100% ET (circles) and 50% ET (triangles) in respective senescence treatments. Sap flow began earlier for trees that had a normal senescence (grey bars) compared to those that had a delayed senescence (dark grey bars) in 2017 (*n* = 6) and 2018 (*n* = 5–6). Early spring sap flow data from the normal senescence trees is missing in 2017 since measurement readings began in April. Error bars represent ± standard error of the mean.

The initiation of spring bloom was significantly delayed on DS trees compared to NS trees in 2017 (*p* ≤ 0.001) and 2018 (*p* ≤ 0.001), with DS trees also showing less flowers than NS trees. The soil moisture treatment had no significant effect on bloom time or the number of flowers in 2017 or 2018. Similarly, spring sap flow began earlier within NS trees in 2016 and 2017 (*p* ≤ 0.001) while DS trees remained dormant longer (Figure 3), but no significant differences were found between the 100% ET and 50% ET trees (data not shown).

## 3. Discussion

Recent studies have shown that an increase in temperature as a result of climate change or climate variability can delay leaf senescence in forest and fruit trees during the fall [28,29]. Similarly, warmer temperatures during fall may result in higher net carbon fixation in deciduous forest trees as a consequence of an extended season of green foliage [15], and relatively high *A* observed in senescing leaves [30]. In our study, warmer temperatures within the greenhouse environment over two fall seasons caused a delay in senescence compared to trees grown at ambient conditions, but leaf *A* of NS and DS trees were similar. The influence of photoperiod, which begins to reduce the photosynthetic capacity after the summer solstice [31] was similar for both DS and NS trees each fall season and may have a stronger influence on fall *A* of young peach trees than temperature and senescence timing. A strong response of the peach trees to photoperiod [32] was evident during the fall of 2016, when lights from a nearby greenhouse delayed the initiation of senescence of both DS and NS trees. 

Analysis of *A_tree_*, using total leaf area in addition to leaf *A*, has been suggested as a measure to determine carbon acquisition and movement within the tree [33]. The leaf area and *A* were similar between NS and DS trees both years of study, suggesting that differences in *A_tree_* may have only occurred at the very end of the growing season, when treatments differed by rates of abscission. Specifically, more leaves were present on DS trees in comparison to NS trees during a period of approximately three weeks (between the end of November and mid-December) in 2016 and five weeks (between mid-October and the end of November) in 2017. Despite these differences in leaf area during the end of each fall season, warmer fall temperatures experienced by DS trees may have caused an increase of respiration [34] and negated any carbon gain due to a delay of senescence. Indeed, the process of cellular breakdown and resorption during senescence has been shown to result in similar, increased, or lower respiration rates when leaves have changed from green to red color [30,35,36] depending on the species examined, and additional measurements of respiration should accompany future work on this topic. Since carbohydrate storage or respiration were not calculated for this study, it was only possible to estimate *A_tree_* differences based on leaf *A* measurements and leaf area. 

Increased temperatures and water stress during fall typically have opposite effects on leaf senescence, with the former typically delaying and the latter advancing the onset of senescence [12]. However, water stress during the summer followed by a recovery period in early fall has been reported to delay senescence in apple trees [29]. In our experiment, water stress started in early fall, and 50% ET trees had lower leaf *A* and a faster defoliation rate than 100% ET trees, although minor compared to the abscission differences caused by the senescence treatment. Furthermore, water stress in fall did not affect spring phenology. Thus, these results indicate that the timing and the balance between temperature and soil moisture factors (or the preponderance of one of them) need to be considered to understand the effects, although different outcomes may occur with other species and climates.

DS trees partitioned less biomass to root growth than NS trees each fall season. Warmer temperature, specifically during the night, inside the greenhouse may have slowed down root growth on DS trees due to higher respiration [14] or growth cessation [37]. Increased R:S ratio have been previously linked to improved water use efficiency (WUE) as a physiological response for better coping with water deficit [38]. The DS trees had both lower leaf WUE (data not shown) and R:S ratio than NS trees, but each of these measurements were taken in different seasons (WUE in fall during gas exchange measurements, R:S ratios in spring) in this study. Additionally, mass and length of fibrous and coarse roots were not explored in this experiment and may elucidate the influence of variable temperature and moisture treatments in future work. The larger R:S ratios during the fall of 2017 may also reflect a generalized water stress on all trees as they became more pot bound the second year of growth and, additionally, were infested with spider mites during the summer months. The lower values of *A* between the early fall of 2017 compared to 2016 were also probably an influence of spider mite damage on all trees. In addition to increasing transpiration and water loss [39] the mites may have accelerated the cellular senescence process on the trees as a result of water stress [12]. Nonetheless, a similar mite presence on the trees did not result in *A* differences between the two senescence treatments.

Spring phenology in DS trees was strongly influenced by warm fall temperatures. With reduced chill accumulation during the end of each fall season inside the greenhouse, the delay of spring flowering of DS trees was expected in comparison to NS trees. In addition to bloom delay, which occurs when peach trees do not fulfill their chilling requirements [40], the extended budbreak observed in DS trees, lasting close to one month for some trees, was probably also a consequence of insufficient chill accumulation. Low chill results in increased heat requirements for floral budbreak, which together with the heterogeneous chilling requirements, even on small trees, helps explain the extended floral budbreak observed [41]. This also matched with the concurrently delayed sap flow movement in spring of the trees with DS in fall. Deducing the effect of fall *A* on the bloom timing is more challenging, as internal signaling from accumulated chill hours probably masked any effect, while any additional carbon gained as a result of DS may have been consumed metabolically prior to budbreak [42]. Regardless, quantification of root respiration and carbon storage during and following DS would allow for exploration of its influence on dormancy break.

In summary, warmer fall temperatures delayed senescence on young peach trees, and although NS and DS trees had comparable rates of leaf *A* during both years of the study, *A_tree_* of DS trees was estimated to be greater since leaves were maintained longer prior to abscission. However, further studies will need to quantify carbon losses through respiration in order to estimate net carbon gain depending on the senescence duration and timing. The influence of warmer fall temperatures also caused a delay in floral budbreak, reduced number of flowers produced, and delayed sap flow the following spring. While 50% ET trees had reduced leaf *A* during the first year of study but not the second, the soil moisture treatments had no significant effect on spring phenology. Changes to phenology during the subsequent year highlights the importance of understanding how variations in climate can influence important perennial crop species.

## 4. Materials and Methods

### 4.1. Study Location, Establishment and Design

This study was carried out under controlled conditions at Clemson University, SC, USA between the fall of 2016 and the spring of 2018. One hundred and twenty-six two-year-old peach trees (at the beginning of the experiment) were grown in 3-gallon pots containing a mix of 2:1 potting soil (Fafard 3B Mix, Sun Gro Horticulture, Agawam, MA, USA): sand, 60 g of lime, and 60 g 14-14-14 slow release fertilizer, and divided into four treatments resulting from a 2 × 2 factorial design comprised of two main effects: (1) Type of senescence: Trees were kept inside a greenhouse during fall (4 °C warmer than ambient outdoor fall temperatures; DS), or trees were placed in a covered structure with ambient outdoor fall temperatures (NS), located adjacent to the greenhouse; and (2) Soil moisture: Trees were either well-watered (100% of their evapotranspirative needs; ET) or water deficient (50% ET) during fall. All treatments included 16 trees except for the 50% ET treatment, which had 15 trees. The four treatments were applied across two cultivars ‘Scarletprince’ and ‘Autumnprince’, which are a mid-season and a late-season cultivar, respectively. While trees were not expected to produce fruit, we intended to evaluate if there would be a cultivar-specific influence on the response to delayed leaf senescence.

In year 1, treatments started in September 2016 and were applied until mid-December. Following the fall season, all trees were watered to field capacity and placed together in outdoor (ambient) temperatures under the covered structure so that leaves would abscise from all the trees regardless of the treatment. In February 2017, eight trees per treatment were harvested for biomass determination (see below), and all other trees remained in the ambient outdoor environment throughout spring and summer until September. In September 2017, the same senescence and irrigation treatments were consecutively applied to the remaining trees for the second year, with trees receiving the same treatment both years. The treatments again concluded in mid-December, and all trees were again watered to field capacity and placed in the outside environment. In February 2018, only four trees per treatment were harvested for biomass determination (through the calculation of their R:S ratio), allowing for spring bloom and sap flow measurements to occur on the remaining trees.

### 4.2. Environmental and Gas Exchange Measurements

Temperature was monitored using WatchDog^®^ (B-Series 102, Spectrum Technologies, Aurora, IL, USA) and Onset^®^ HOBO^®^ data loggers (MX220, Onset Computer Corporation, Bourne, MA, USA). The minimum temperature of the greenhouse environment had a lower setting for the 2016 fall (17 °C) in comparison to 2017 (25 °C), however the greenhouse environment was on average 4 °C warmer than the ambient outdoor environment each fall season. Artificial lights from an adjacent greenhouse were a source of evening light for both the greenhouse and outdoor locations in 2016 while black plastic curtains were erected to block this additional light in 2017.

Whole tree ET was gravimetrically calculated similarly to Romero-Conde et al. [43], with weight measurements of the pots being made on two consecutive days twice each year (September 15–16 and November 15–16 in 2016, and September 9–10 and October 10–11 in 2017). Following the completion of each fall season in December and after all trees were watered to field-capacity, no irrigation occurred until growth resumed at bud-swell in spring. 

A portable photosynthesis system (LICOR 6400-XTR, Li-COR, Lincoln, NE, USA) was used to measure tree gas exchange parameters including *A* (μmol [CO_2_] m^−2^ s^−1^), leaf-to-air vapor pressure difference (VPD [kPa]), and leaf transpiration rate (E [mmol (H_2_O) m^−2^ s^−1^]) to calculate leaf WUE (mmol [CO_2_] mol [H_2_O]^−1^]). Fully developed leaves (4–5th node) from six different trees per treatment were used for these measurements at each sampling date in 2016 (September 30, October 13, October 31, November 14, December 13) and 2017 (September 25, October 10, October 24, November 9, November 21). Measurements were taken from 9:00 AM to 12:00 PM at a photosynthetic photon flux density of 1000 µmol m^−2^ s^−1^ and 400 ppm atmospheric CO_2_. The VPD values were different between NS and DS trees during each sampling period both years (Table 2). *A_tree_* was estimated as in Pornon and Lamaze [44] by multiplying the average leaf *A* at each sampling date with the average leaf area and number of leaves per treatment. Estimations of *A_tree_* were started each year when leaf color began to visually change before leaf fall (29 November 2016 and 16 October 2017), and leaf area was measured once with a LI-3100 leaf area meter (Li-COR, Lincoln, NE, USA) on 7 October 2016 using 50 leaves per treatment.

### 4.3. Biomass and Spring Phenology

The influence of a longer growing season on carbon partitioning was explored by performing biomass determinations in the late winter following each fall season. In February, trees were cut apart, roots and shoots were separated, and soil was carefully removed from roots. Both roots and shoots were cut into pieces and oven-dried for two weeks at 70 °C before measuring dry mass. Specifically in 2017, 16 trees per treatment were measured to calculate biomass, while only eight trees per treatment were measured in 2018 as the remaining trees were kept for bloom and sap flow measurements.

The total number of open blooms with petals still attached were counted from 16 trees every four days during the spring of 2017 and from 8 trees in 2018. Sap flow was measured every 30 min using sap flow sensors (Model SGEX25 and Flow32-1K monitoring system, Dynamax, Houston, TX, USA) attached to six NS and six DS trees during spring (3 April through 1 June 2017, and 20 February through 30 May 2018). In 2018, one NS tree died and was excluded. 

### 4.4. Statistical Analysis

The experiment followed a 2 × 2 factorial design with type of senescence and soil moisture as the two factors. The cultivar had no effect on leaf *A* or spring phenology and was excluded as a factor. Differences between senescence and soil moisture and their interaction regarding leaf *A* and *A_tree_*, spring flowering, biomass, and sap flow were analyzed using analysis of variance. Mean separation was performed using Student’s least significant difference post hoc test. All data were analyzed using JMP^®^ statistical software (Version 14.1.0; SAS institute, Cary, NC, USA).

## Figures and Tables

**Table 1:** Mean root to shoot (R:S) ratios of peach trees harvested during the winter of 2017 (*n* = 16) and 2018 (*n* = 8) after the treatments: (1) Delay of senescence (DS) or normal senescence (NS) and (2) receiving the total daily evapotranspirative needs (100% ET) or half (50% ET) the previous fall.

Treatment	2017		2018	
	R:S	*p*	R:S	*p*
NS	0.53 a	<0.01	0.84 a	0.08
DS	0.43 b		0.75 a	
100% ET	0.48 a	0.95	0.78 a	0.94
50% ET	0.48 a		0.79 a	

Different letters indicate differences using Student’s least significant difference mean separation test (α = 0.05) by treatment within each year.

**Table 2:** Average vapor pressure difference (VPD) values (kPa ± SE) during each sampling period in 2016 and 2017 between normal senescence (NS; ambient outdoor environment) and delayed senescence (DS; greenhouse) trees (*n* = 40–44). For each date, different letters indicate significant differences using Student’s least significant difference mean separation test (α = 0.05).

Year and Treatment		Date and VPD (kPa)				
2016		September 30	October 13	October 31	November 14	December 13
	NS	2.32 ± 0.06 b	2.02 ± 0.07 b	2.12 ± 0.07 b	1.46 ± 0.05 b	1.24 ± 0.02 a
	DS	2.80 ± 0.07 a	2.47 ± 0.09 a	2.68 ± 0.07 a	2.00 ± 0.06 a	1.00 ± 0.02 b
2017		September 25	October 10	October 24	November 9	November 21
	NS	2.53 ± 0.08 a	2.43 ± 0.02 b	1.89 ± 0.06 b	1.23 ± 0.04 b	1.74 ± 0.03 b
	DS	1.89 ± 0.07 b	2.52 ± 0.02 a	2.25 ± 0.06 a	1.78 ± 0.05 a	1.92 ± 0.03 a
